# *Gfi1* and *Gfi1b* Repress *Rag* Transcription in Plasmacytoid Dendritic Cells *In Vitro*

**DOI:** 10.1371/journal.pone.0075891

**Published:** 2013-09-24

**Authors:** Kwan T. Chow, Danae Schulz, Sarah M. McWhirter, Mark S. Schlissel

**Affiliations:** 1 Department of Molecular & Cell Biology, University of California, Berkeley, California, United States of America; 2 Office of the Provost, Brown University, Providence, Rhode Island, United States of America; Klinikum rechts der Isar der TU München, Germany

## Abstract

*Growth factor independence* genes (*Gfi1* and *Gfi1b*) repress recombination activating genes (Rag) transcription in developing B lymphocytes. Because all blood lineages originate from hematopoietic stem cells (HSCs) and different lineage progenitors have been shown to share transcription factor networks prior to cell fate commitment, we hypothesized that GFI family proteins may also play a role in repressing *Rag* transcription or a global lymphoid transcriptional program in other blood lineages. We tested the level of *Rag* transcription in various blood cells when *Gfi1* and *Gfi1b* were deleted, and observed an upregulation of *Rag* expression in plasmacytoid dendritic cells (pDCs). Using microarray analysis, we observed that *Gfi1* and *Gfi1b* do not regulate a lymphoid or pDC-specific transcriptional program. This study establishes a role for *Gfi1* and *Gfi1b* in *Rag* regulation in a non-B lineage cell type.

## Introduction

*Gfi1* and *Gfi1b* encode 2 highly homologous nuclear proteins that function as transcriptional repressors. These proteins share a conserved C-terminal domain containing 6 zinc finger motifs that mediate DNA binding activity, and an N-terminal SNAIL/GFI-1 (SNAG) domain that mediates association with chromatin modifiers with repressive function [1-3]. *Gfi1* and *Gfi1b* are widely expressed in the hematopoietic system [4,5]. They are both expressed in hematopoietic stem cells (HSCs) and common lymphoid progenitors (CLPs), as well as early B and T cells. *Gfi1* is expressed in the monocytic and granulocytic lineages, while *Gfi1b* is expressed in megakaryocytic and erythrocytic lineages [6].

GFI1 and GFI1B are crucial transcriptional regulators during hematopoiesis, and play important roles in multi-lineage blood cell development [7]. Both proteins are important factors for the endothelial-to-hematopoietic transition during HSC generation, and both have been shown to restrict HSC proliferation. *Gfi1* also functions to maintain self-renewal capacity and engraftment of HSCs [8]. In the myeloid compartment, *Gfi1* orchestrates the linage fate decision between monocytes/macrophages and granulocytes [9]. *Gfi1* deficient mice lack neutrophils, and accumulate a population of morphologically atypical immature monocytes that have the potential to generate mature macrophages but fail to produce granulocytes. Furthermore, development of dendritic cells (DCs) also depends on the expression of *Gfi1*, as mice lacking this protein show defective DC maturation and an overabundance of macrophages. In the lymphoid compartment, *Gfi1* is important for both B and T cell development. *Gfi1* deficient mice have significantly reduced numbers of B cells, and exhibit decreased thymic cellularity due to reduced proliferation, increased apoptosis and an early block at the DN stage of T cell development [10]. The exact role of *Gfi1b* in hematopoiesis is less well established because *Gfi1b* deficiency in mice results in embryonic lethality at E15 [6]. These animals likely die of failure to develop red blood cells, implicating a crucial role for *Gfi1b* in erythropoiesis. *Gfi1b* knockout mice also fail to develop megakaryocytes, but have arrested erythroid and megakaryocytic precursors in the fetal liver. *In vitro*, overexpression of *Gfi1b* inhibits myeloid differentiation of a cultured myelomonocytic cell line [11]. Recent generation of a conditional knockout model of *Gfi1b* has enabled analysis of the specific function of *Gfi1b* in adult hematopoiesis. It has been shown that B cell specific *Gfi1* and *Gfi1b* double knockout mice have an exacerbated phenotype as compared to the *Gfi1* single knockout and fail to generate any B cells [12]. This mouse model will continue to be an ideal tool to dissect the specific function of *Gfi1b* in different hematopoietic lineages.

Recently, we identified *Gfi1* and *Gfi1b* as transcriptional repressors of the V(D)J *recombination* activating genes, *Rag1* and *Rag2* (collectively known as *Rag*), during B cell development [12]. Because *Rag* expression is largely lymphoid restricted, we asked whether *Gfi1* and *Gfi1b* may play a role in repressing *Rag* expression in other blood lineages, which often share common transcription factor networks [13]. Furthermore, because GFI family proteins play important roles in cell fate decision during hematopoiesis, we hypothesized that they may also be responsible regulating a global lymphoid transcriptional program.

We utilized a V(D)J recombination reporter system [14] to monitor RAG activity during multi-blood lineage differentiation *ex vivo* when *Gfi1* and *Gfi1b* were simultaneously deleted. We found that deletion of these genes resulted in upregulation of *Rag* expression in plasmacytoid dendritic cells (pDCs), but not in other blood lineages tested. However, while these *Gfi1* and *Gfi1b* have diverse gene targets, they do not appear to regulate a lymphoid-specific transcriptional program. Our data revealed a novel role of *Gfi1* and *Gfi1b* in *Rag* repression in a non-B blood lineage cell type.

## Results

### Deletion of *Gfi1* and *Gfi1b* increases expression of a V(D)J recombination reporter in plasmacytoid dendritic cells *in vitro*

Because *Gfi1* and *Gfi1b* repress *Rag* transcription in developing B cells [12], we hypothesized that they may also play a role in repressing *Rag* expression in non-lymphoid blood lineages that share common transcription factor networks [13]. To test this hypothesis, we utilized the H2-SVEX reporter mouse to detect RAG activity in non-B lineage cells. The H2-SVEX mouse carries a transgene expressing a violet light excited (VEX) fluorescent protein cDNA in the antisense orientation driven by a promiscuously active promoter. The cDNA is flanked by V(D)J recombination signal sequences (RSSs) oriented such that V(D)J recombination results in an inversion of the VEX cDNA into the sense orientation, irreversibly marking cells that have experienced *Rag* activity [14]. We generated a mouse carrying the H2-SVEX transgene and an ERT2-Cre cDNA knocked into the *Rosa26* locus [15], that was also homozygous for floxed alleles of *Gfi1* and *Gfi1b* [16,17]. The encoded ERT2-Cre protein allows for tamoxifen-inducible deletion of *Gfi1* and *Gfi1b*.

**Figure 1:**
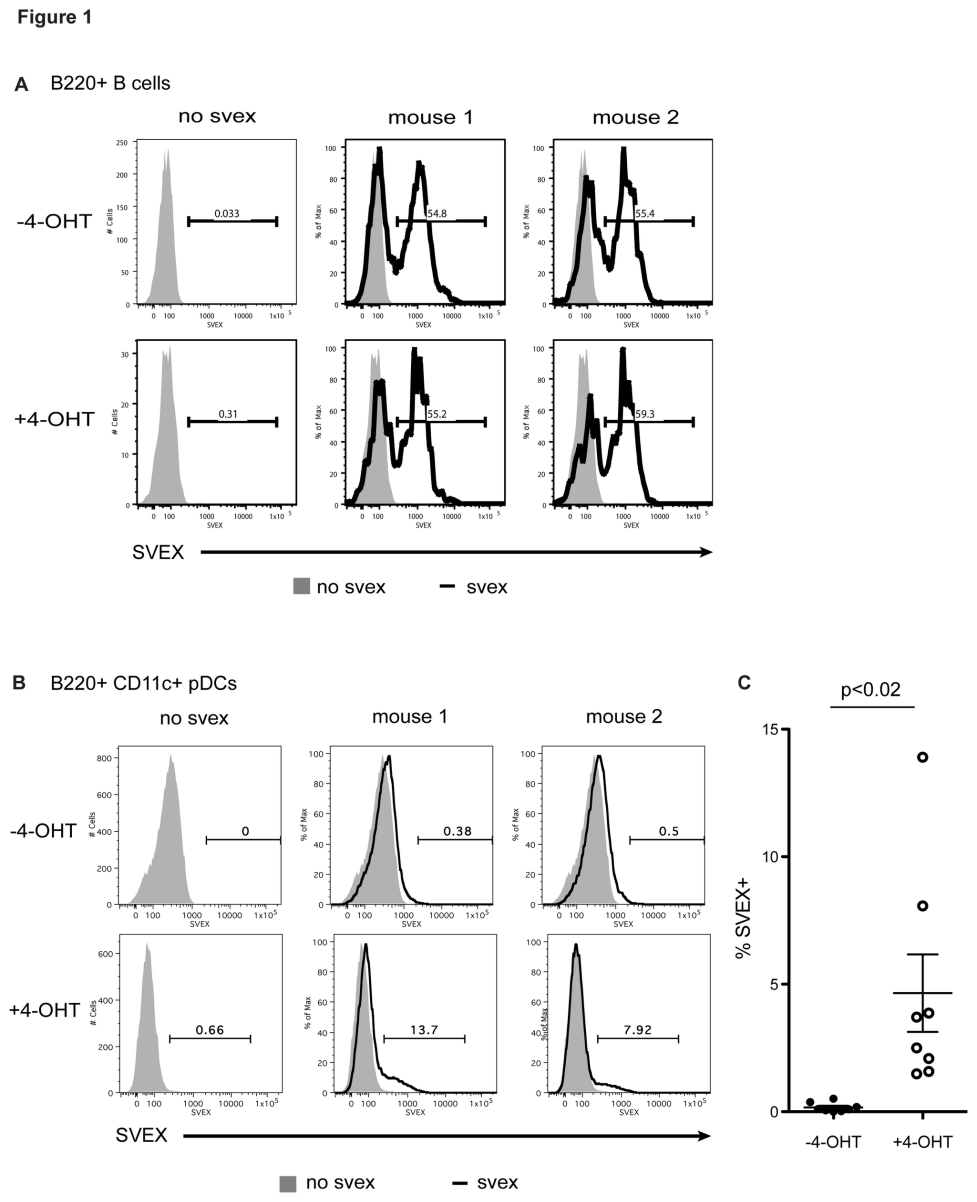
Deletion of *Gfi1* and *Gfi1b* results in increased V(D)J recombination in B cells and pDCs. (**A**) Flow cytometric analysis of VEX expression in B cells derived from the bone marrow of 2 individual *Gfi1* ^*f/f*^ *; Gfi1b* ^*f/f*^ *; ERCre, SVEX* mice cultured in 5ng/ml IL-7 for 7 days (solid line), untreated (top panel) and treated (bottom panel) with tamoxifen (4-OHT). Cells were gated on B220^+^. Shaded histogram denotes background fluorescence from *Gfi1* ^*f/f*^ *; Gfi1b* ^*f/f*^ *; ERCre* cells. Vertical axis ('% of max') indicates a scale of relative cell numbers with the median value set as 100%. (**B**) Flow cytometric analysis of VEX expression in pDCs derived from bone marrow from 2 individual *Gfi1* ^*f/f*^ *; Gfi1b* ^*f/f*^ *; ERCre, SVEX* mice cultured in 25ng/ml Flt-3L for 8 days (solid line), untreated (top panel) and treated (bottom panel) with tamoxifen. Cells were gated on B220^+^ CD11c^+^ cells. Shaded histogram denotes background fluorescence from *Gfi1* ^*f/f*^ *; Gfi1b* ^*f/f*^ *; ERCre* cells. Vertical axis ('% of max') indicates a scale of relative cell numbers with the median value set as 100%. All data are representative of at least three independent experiments. (**C**) Dot plot showing percentage of SVEX+ cells in Flt-3L cultures untreated (-4-OHT) and treated (+4-OHT) with tamoxifen. p-value was calculated using the two-tail paired Student’s *t*-test.

We opted for an *ex vivo* system to test whether *Gfi1* and *Gfi1b* repress *Rag* expression in non-lymphoid blood lineages because *Gfi1* and *Gfi1b* deficiency results in cell lethality in multiple blood lineages *in vivo* [6,10,18-21]. Using established cytokine-driven culture systems, we differentiated bone marrow progenitor cells from this mouse (*Gfi* ^f/f^, *Gfi1b* ^f/f^, ERT2-Cre, SVEX) into macrophages, natural killer (NK) cells, megakaryocytes, conventional dendritic cells (cDCs) and plasmacytoid dendritic cells (pDCs) [22-26]. During differentiation, we treated half the culture with tamoxifen to delete *Gfi1* and *Gfi1b* (KO), and left the other half untreated (WT). We then assayed for VEX expression in these cultures by flow cytometry. Since we observed that the background fluorescence levels differed in different culture conditions, we used a mouse of the same genotype but lacking the H2-SVEX transgene as a negative control. As expected, VEX expression was readily detected in *ex vivo* differentiated B cells, indicating that the reporter faithfully reflects *Rag* expression in culture (Figure 1A). We noted that VEX expression did not increase in progenitor B cell cultures treated with tamoxifen, suggesting that either the expected increase in *Rag* levels due to deletion of *Gfi1* and *Gfi1b* is not sufficient to increase recombination, or that the recombination of H2-VEX transgene is not 100% efficient. In fact, both in our hands and in published data, only 50-85% of splenic B cells express VEX, whereas 100% of them have a history of *Rag* expression [27].

We could not detect VEX expression in *ex vivo* differentiated macrophages, NK cells, megakaryocytes or cDCs, either in WT and KO cultures (data not shown). In pDC cultures, however, we detected 3-15% SVEX+ cells in the tamoxifen-treated cultures but not in untreated cultures (Figure 1B). The difference in SVEX+ cells is statistically significant between cultures untreated and treated with tamoxifen (Figure 1C). These data suggest that deletion of *Gfi1* and *Gfi1b* leads to aberrant V(D)J recombination activity in pDCs.

### GFI proteins regulate *Rag* expression in plasmacytoid dendritic cells

**Figure 2:**
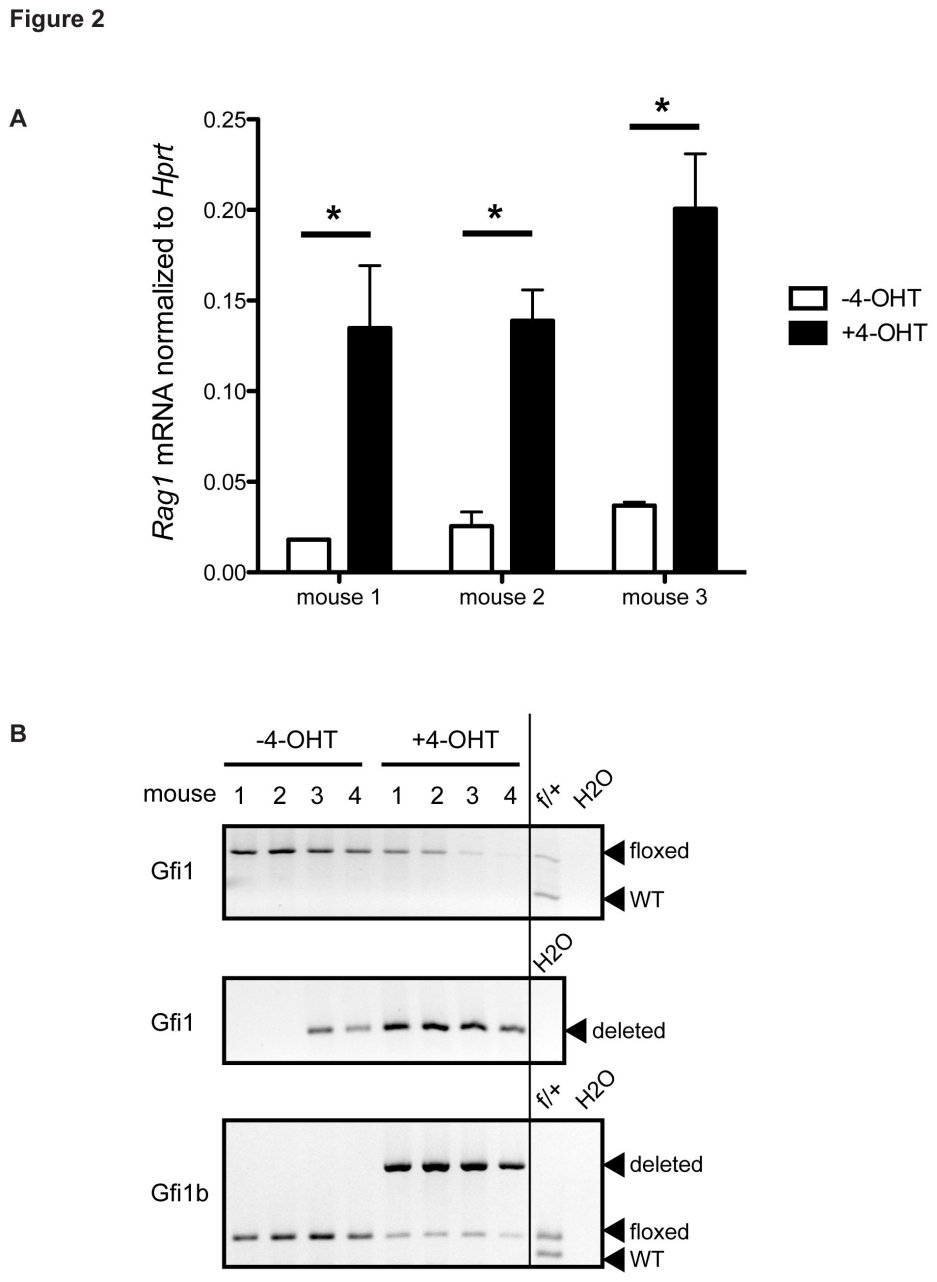
Deletion of *Gfi1* and *Gfi1b* results in increased expression of *Rag* in pDCs. (**A**) Quantitative RT-PCR analysis of *Rag1* transcript levels in sorted B220^+^ CD11c^+^ pDCs derived from 3 individual *Gfi1* ^*f/f*^ *; Gfi1b* ^*f/f*^ *; ERCre* mice untreated and treated with tamoxifen (4-OHT). Values are normalized to *Hprt1* transcript abundance. p-values were calculated with the two-tail Student’s *t*-test. * denotes p < 0.005. (**B**) Genotyping PCR of *Gfi1* and *Gfi1b* loci from sorted B220^+^ CD11c^+^ pDCs derived from 4 individual *Gfi1* ^*f/f*^ *; Gfi1b* ^*f/f*^ *; ERCre* mice untreated and treated with tamoxifen (4-OHT). Genomic DNA was isolated and subjected to PCR analysis using primers that detect wildtype (WT), floxed and deleted alleles of *Gfi1* and *Gfi1b*. PCR products were run on 1% agarose gel and visualized with ethidium bromide. All data are representative of at least two independent experiments.

To confirm that the VEX expression in pDC cultures was indeed due to misregulated *Rag* expression, we sorted *ex vivo* differentiated pDCs using CD11c and B220 surface markers (Figure S1), and measured *Rag* expression by quantitative realtime PCR (RT-qPCR). As compared to pDCs derived in untreated cultures, tamoxifen-treated pDCs showed a 2-3 fold increase in *Rag* expression (Figure 2A), which strongly correlated with the increase in VEX expression in these cultures. The degree of de-repression was similar to that in B cells when *Gfi1* and *Gfi1b* were deleted [12]. We observed that tamoxifen-induced deletion of *Gfi1* and *Gfi1b* in pDC cultures was quite inefficient as assayed by genotyping PCR (Figure 2B), suggesting that the observed increase in *Rag* expression upon *Gfi1* and *Gfi1b* deletion in these cultures was likely an underestimate.

### GFI proteins do not repress expression of other lymphoid genes in plasmacytoid dendritic cells

Because *Rag* expression is generally restricted to the lymphoid lineage, we next asked whether other lymphoid-specific genes were also regulated by GFI proteins in pDCs. We purified RNA from sorted *ex vivo* differentiated pDCs from untreated (WT) and tamoxifen-treated (KO) cultures and performed a microarray analysis to obtain a global view of their gene expression landscapes. We used GenePattern [28] to identify a set of genes that are differentially expressed in WT and KO pDCs by at least 2 fold (Table S1). This set of genes is not lymphoid-specific, but includes genes regulating diverse cellular processes, including cell adhesion, cytokine signaling, chemotaxis, and differentiation.

**Figure 3:**
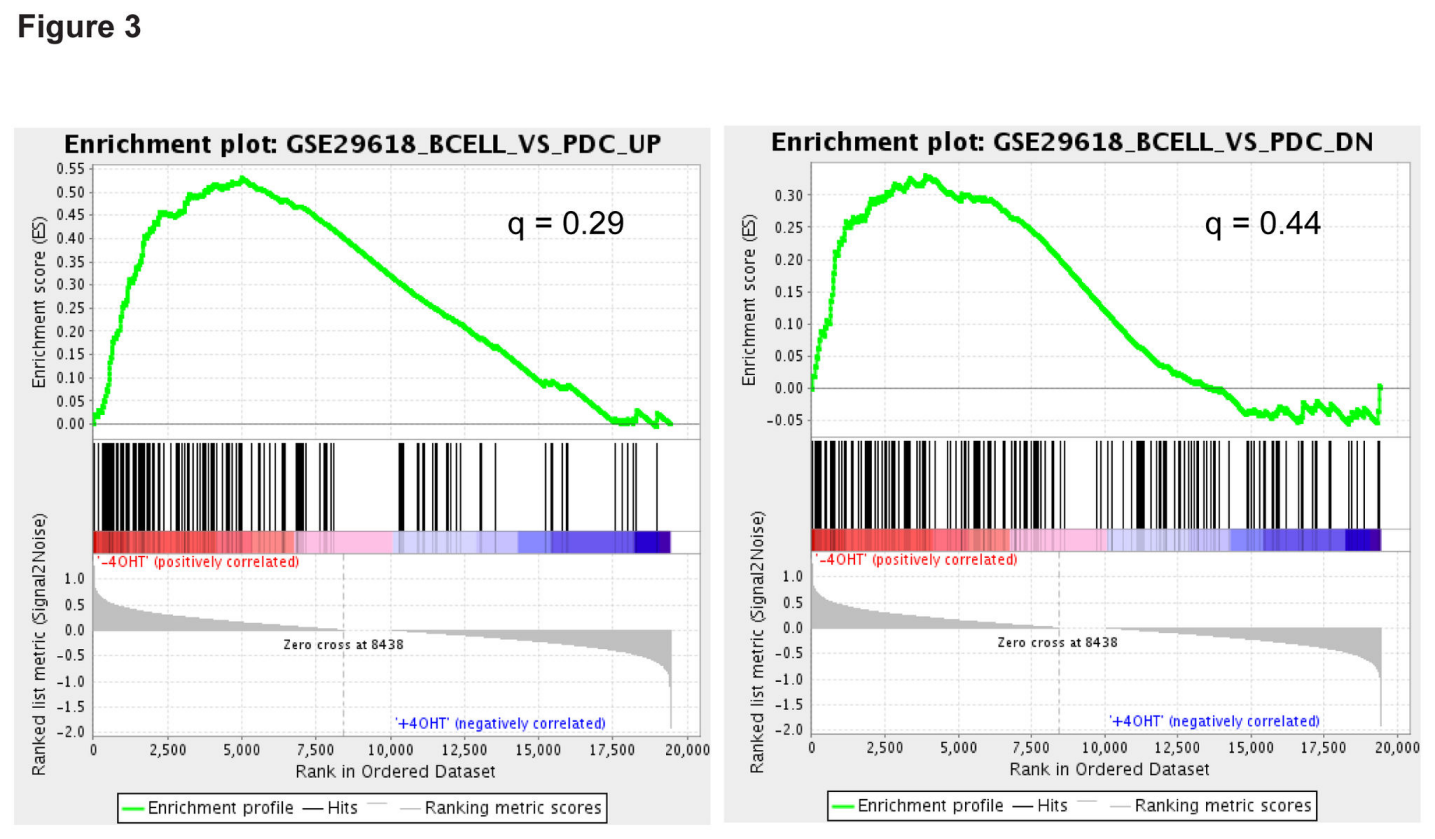
Deletion of *Gfi1* and *Gfi1b* does not result in misregulation of a global lymphoid or pDC program in pDCs. Gene Set Enrichment Analysis (GSEA) was performed with the GenePattern platform. GSE29618_BCELL_VS_PDC_UP and GSE29618_BCELL_VS_PDC_DN gene sets were tested for enrichment in WT and KO pDC cultures. False discovery rate (FDR) q-values indicate the likelihood of false enrichment.

To ask whether deleting GFI proteins in pDC cultures results in a global change in lymphoid- or pDC-specific genes, we performed gene set enrichment analysis (GSEA) using curated gene sets available in the Molecular Signature Database (MSigDB) [29]. We used the GSE29618 gene sets in the C7 Immunologic Signature collection, which comprises of a set of upregulated and a set of downregulated genes when comparing gene expression profiles of B vs. pDCs [30]. We asked whether these gene sets are enriched in WT or KO pDC cultures using the false discovery rate (FDR) cutoff of 25%, a standard cutoff for GSEA analysis indicating that the probability of a false enrichment is below 25% [29]. This is represented by the q-value of < 0.25 [29]. While both gene sets showed a positive enrichment score (positively correlated with WT cultures), neither gene sets passed the FDR cutoff (Figure 3). Further, we also generated and tested gene sets that are upregulated and downregulated in B cells or pDCs when compared to HSCs using transcriptional profiling data generated by the Immunological Genome Project [31] (Table S2). We again found no statistically significant enrichment of the gene sets tested (data not shown). These results indicate that deleting GFI proteins in pDCs does not result in a global change in lymphoid- or pDC- specific genes.

**Figure 4:**
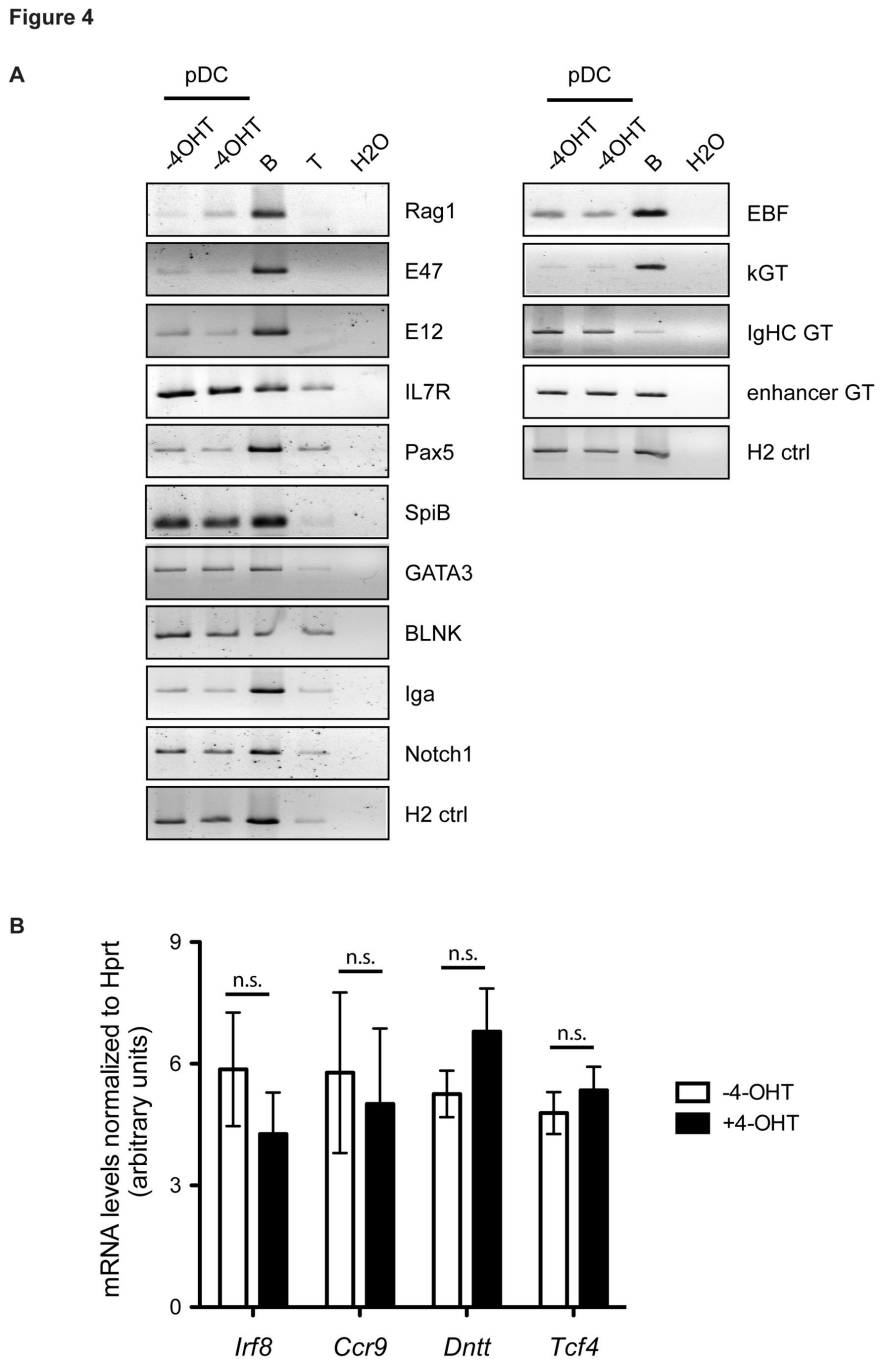
Deleting *Gfi1* and *Gfi1b* does not result in a change in lymphoid- or pDC-specific genes in pDCs. (**A**) RT-PCR of lymphoid-specific gene expression in *ex* *vivo* differentiated B220^+^ CD11c^+^ pDCs from *Gfi1* ^*f/f*^ *; Gfi1b* ^*f/f*^ *; ERCre* mice untreated (WT) and treated (KO) with tamoxifen. RNA isolated from primary B (B) or T (T) cells were used as controls. (**B**) Quantitative RT-PCR of pDC-specific genes in *ex* *vivo* differentiated B220^+^ CD11c^+^ pDCs from *Gfi1* ^*f/f*^ *; Gfi1b* ^*f/f*^ *; ERCre* mice untreated (WT) and treated (KO) with tamoxifen. Expression values in arbitrary units are normalized to *Hprt* transcript abundance.

We noted that *Rag* and many lymphoid genes had low expression levels in WT pDCs. In fact, the list of genes found to be differentially expressed in WT vs. KO *ex vivo* differentiated pDCs did not include *Rag* because of the stringent thresholding and cutoff criteria, even though increase in *Rag* expression in KO cultures were confirmed by RT-qPCR before samples were subjected to microarray analysis (data not shown). To ensure that our global gene expression analysis did not miss subtle changes in the expression of individual lymphoid genes, we purified RNA from sorted *ex vivo* differentiated pDCs from untreated and tamoxifen-treated cultures and measured expression levels of individual genes by RT-PCR. We tested a set of lymphoid genes normally expressed in wildtype primary pDCs, including *Rag* [32]. We detected no increase in expression of any of the lymphoid genes tested except for *Rag* (Figure 4A).

To further investigate whether GFI proteins regulate pDC-specific genes, we measured the expression of several key pDC-specific genes in WT and KO pDC cultures by quantitative RT-PCR. Two transcription factors, important for pDC development (*Irf8* and *Tcf4*), as well as two lymphoid genes that are also highly expressed in pDCs (*Dntt* and *Ccr9*), showed no significant differences in expression in WT vs. KO pDCs (Figure 4B). Taken together, the results in Figure 4 further validate the findings of our microarray analysis, and suggest that outside the B cell lineage, GFI proteins can regulate expression of *Rag*, as well as a diverse set of genes, but not a global lymphoid or pDC-specific transcriptional program.

## Discussion

GFI1 and GFI1B are crucial transcriptional regulators during hematopoiesis. Mouse models in which a GFP cDNA was knocked into the *Gfi1* or *Gfi1b* loci have shown that these genes are widely expressed within the hematopoietic system [4,5]. They are essential for development of multiple blood lineages as mice deficient of *Gfi1* or *Gfi1b* have significant defects in hematopoiesis [33]. We previously identified these proteins as repressors of *Rag* expression in developing B cells [12]. In this study, we demonstrated that these proteins also repress *Rag* expression in plasmacytoid dendritic cells (pDCs) *in vitro*. They, however, do not orchestrate a global lymphoid or pDC-specific transcriptional program, but regulate diverse set of genes during pDC development.

*Gfi1* and *Gfi1b* are paralogs with very similar structures. They share conserved N-terminal and C-terminal domains, but variable intermediate region. Association of GFI1 and GFI1B with chromatin modifiers through their N-terminal SNAG domains allows them to reversibly repress their targets [3]. While it has been proposed that specific target genes may exist for GFI1 and GFI1B, both proteins share overlapping targets and exhibit functional redundancy, especially during hematopoiesis [34]. Indeed, we observed that single deletion of either *Gfi1* or *Gfi1b in vivo* does not alter the level of *Rag* transcription in developing B cells, but deletion of both proteins simultaneously results in misregulation of *Rag* transcription in B cells [12]. In this study, we showed that deleting both *Gfi1* and *Gfi1b* results in an increase in *Rag* expression in pDCs to an extent similar to that in B cells (2-3 fold). It is interesting to note that *Gfi1*-deficiency results in a 50% reduction in the numbers of pDC *in vivo* [35], implicating a role for GFI proteins in pDC development. We did not observe aberrant *Rag* expression in other cell types tested. However, we cannot exclude the possibility that deletion of *Gfi1* and *Gfi1b* may affect survival of certain cell types, thus hindering the analysis of their specific function in *Rag* repression in these cell types.

Because *Gfi1* and *Gfi1b* have been shown to be important for the differentiation of multiple blood lineages, we hypothesized that they may play a broader role beyond repressing *Rag* expression. All blood lineages originate from the hematopoietic stem cells (HSCs), which give rise to multi-potent progenitors (MPPs). These progenitors share transcription factor networks prior to commitment and restriction to a specific cell fate [36]. This phenomenon is termed transcriptional priming, and likely reflects the plasticity and the multi-lineage generation capacity of MPPs on a molecular level. Specification of cell fate thus requires the resolution of a mixed lineage gene expression pattern by induction and repression of lineage-specific genes [37-40]. Because *Gfi1* and *Gfi1b* are crucial regulators of hematopoiesis, we postulated that they may play a role in transcriptional priming. Indeed, *Gfi1* has been shown to be a direct downstream target of Ikaros, a key regulator of lymphoid priming during early hematopoiesis [41,42]. *Gfi1* is part of a regulatory network that determines lineage fate decision between granulocyte and monocyte/macrophage development by antagonizing PU.1, another key factor for lineage-specific hematopoietic differentiation [42,43]. However, our microarray results suggest that these proteins play little role in specifying a lymphoid or pDC-specific transcriptional program in pDCs. While it is clear that these proteins regulate vast numbers of genes as previously shown, the gene targets are not specific to a certain lineage. Together, these data suggest that *Gfi1* and *Gfi1b* participate in many cellular functions in pDCs, but do not regulate a lymphoid or pDC-specific gene expression profile.

Our data indicate that GFI1 and GFI1B are negative regulators of *Rag* in pDCs, but not in other cell types tested. Wildtype pDCs have been shown to express low levels of *Rag*, as well as a global lymphoid-like transcriptional program [44]. Lineage tracing experiments showed that 20-30% of pDCs have a history of *Rag* expression in mice [45,46]. This is believed to be an indication of the lineage affiliation of pDC development. While pDCs are clearly affiliated with the dendritic cell lineage, they show genetic and functional overlap with B cells [47]. Common lymphoid progenitors (CLPs) are capable of giving rise to pDCs [48], and pDC development is dependent on transcription factors that are also essential for B cell development, such as Ikaros, SpiB and E proteins [49-52]. Besides a set of lymphoid-specific genes including *Rag*, *Dntt* and *VpreB* [32], pDCs also express CD45R/B220, a B cell-specific surface marker [53]. *Rag* expression in pDCs is functional, as pDCs undergo partial (D-J) rearrangement at the immunoglobulin heavy chain locus [32], a hallmark of early developing B cells. Moreover, the BDCA2 receptor on pDCs has been shown to signal through signaling components of the B cell receptor, including Syk and SLP-65 [54,55]. The namesake refers to the “plasmacytoid” secretory morphology of pDCs that resembles antibody-secreting plasma B cells, and the localization and homing pattern of pDCs also resembles that of B cells [56]. These characteristics indicate that pDCs host a lymphoid-like molecular environment that is permissive to *Rag* expression. We set out to test the hypothesis that GFI proteins are master regulators of *Rag* expression, without which aberrant *Rag* expression would occur in all cell types. Our data, however, support a different model, where GFI proteins are acting as dampers instead of OFF-switches. This model suggests that most cell types have other robust mechanisms to suppress *Rag* expression, thereby preventing genomic instability. Thus, deleting *Gfi1* and *Gfi1b* would not be predicted to alter *Rag* expression. However, in an environment permissive to *Rag* expression, such as in B cells or pDCs, GFI proteins keep *Rag* levels from being dangerously high. We postulate that the observed 3-15% of pDCs that aberrantly expressed *Rag* upon GFIs deletion were likely pDCs that already expressed *Rag* (20-30% of pDCs). This study demonstrates a new role for GFI proteins in regulating *Rag* expression in pDCs, and at the same time reveals the complex layers of regulation of *Rag* expression in different blood lineages.

## Materials and Methods

### Ethics statement

All mouse experimentation was approved by the Animal Care and Use Committee of the University of California, Berkeley (Protocol # R253-0313BR). The handling of the animals was in accordance with this protocol.

### Mice

*Gfi1* ^*f/f*^ and *Gfi1b* ^*f/f*^ mice are kindly provided by Dr. Tarik Moroy (University of Montréal). H2-SVEX mice are kindly provided by Dr. Rachel Gernstein (University of Massachusetts). ER-Cre mice were obtained from Jackson Laboratory (Bar Harbor, ME).

### Chemicals

4-hydroxy-Tamoxifen was purchased from Calbiochem. Recombinant mouse IL-7 and Flt-3 ligand were purchased from R&D Systems.

### Ex vivo differentiation

Total bone marrow was obtained from flushing femurs and tibias from *Gfi1* ^*f/f*^; *Gfi1b* ^*f/f*^; *ERCre*; *SVEX* mice. Cells were cultured in RPMI 1640 medium supplemented with 10% (vol/vol) FCS, L-glutamine (2 mM), penicillin (100 g/ml), streptomycin (100 g/ml) and 2-mercaptoethanol (50 mM). For B cell cultures, media was supplemented with 5ng/ml IL-7. For pDC cultures, media was supplemented with 25ng/ml Flt-3L. All cells were grown at 37°C in 5% CO_2_. Cells were stained and analyzed by flow cytometry 7-8 days post differentiation.

### Flow cytometry

Single-cell suspensions were prepared from mice or from cultured cells and were labeled with fluorochrome-conjugated antibodies by standard techniques. A FC500 (Beckman Coulter) or LSRII (BD Biosciences) flow cytometer was used for analysis; a MoFlo or an Influx high-speed cell sorter (Dako-Cytomation) was used for sorting. Data were analyzed with FlowJo software (Tree Star). Dead cells were gated out using forward and side scatter for all analyses. Analysis with *ex vivo* differentiated B cells was done by labeling cells with anti-B220 (RA3-6B2) antibody. Analysis with *ex vivo* differentiated pDCs was done by labeling cells with anti-B220 and anti-CD11c (N418) antibodies. Anti-B220 and anti-CD11b antibodies were obtained from eBioscience. Statistical significance was performed using two-tail paired Student’s *t*-test.

### Genotyping PCR

Genomic DNA was isolated by phenol/chloroform extraction. PCR was performed with house-made Taq polymerase under cycling conditions of 95°C for 2 min, followed by 32 cycles of 95°C for 40 sec, 60°C for 40 sec and 72°C for 40 sec. Primers sequences are provided in Table S3.

### Gene expression analysis by RT-PCR or quantitative real-time PCR

RNA was isolated by lysing cells in TRIzol reagent (Invitrogen). Reverse transcription was performed using MMLV-RT (Invitrogen) or SuperScript III-RT (Invitrogen) with random hexamers according to the manufacturer’s instructions. Quantitative real-time PCR was performed using JumpStart Taq polymerase (Sigma) according to the manufacturer’s protocol and fluorescent labeling with EvaGreen (Biotium). PCR cycling conditions were 95°C for 4 min followed by 40 cycles of 95 °C for 30 sec and 60 °C for 30 sec. Statistical analysis was performed using the two-tail paired Student’s *t*-test. RT-PCR was performed with house-made Taq with cycling condition of 95°C for 2 min followed by 32 cycles of 95°C for 30 sec, 60°C for 30 sec and 72°C for 40 sec. Primer sequences are provided in Table S3.

### Microarray analysis

Tamoxifen-treated and untreated *ex vivo* differentiated pDCs from 3 independent *Gfi1* ^*f/f*^ *; Gfi1b* ^*f/f*^ *; ERCre* mice were collected by sorting for B220 ^+^ CD11c^+^ cells. RNA was isolated with TRIzol reagent (Invitrogen), and further purified by RNeasy Mini kit (Qiagen). Samples were submitted for analysis to the UC, Berkeley QB3 functional genomics core facility. Affymetrix GeneChip Mouse Gene 1.0 ST Arrays (cat# 901169) were used. Differential gene expression analysis was performed using GenePattern platform (http://www.broadinstitute.org/cancer/software/genepattern/). Microarray dataset was deposited to NCBI GEO repository (GSE45837).

### Bioinformatics

GSEA analysis was performed with gene sets available in the MSigDB database v4.0 curated by the GSEA Team at the Broad Institute (http://www.broadinstitute.org/gsea/msigdb/index.jsp). Analysis was performed using the GSEA module on the GenePattern platform. Custom gene sets comparing B vs. HSC and pDC vs. HSC were generated from publically available gene expression profiles of primary B, pDCs and HSCs from the ImmGen database (www.immgen.org).

## Supporting Information

Figure S1 **Gating strategy of pDC cultures.** Representative gating strategy of flow cytometric analysis of pDCs derived from FLT-3L cultures. Cultures were untreated (top panel) and treated (bottom panel) with tamoxifen (4-OHT). Cells were gated on FS/SS, then CD11c^+^, then B220^+^.
(TIF)

Table S1 **Genes differentially expressed in WT vs. KO pDC cultures from microarray analysis.**
(XLSX)

Table S2 **Gene lists created by comparing gene expression profiles of HSC, B and pDC, used for GSEA analysis.**
(XLSX)

Table S3 **Primer sequences used in this study.**
(DOCX)
