# Supplementary figures and images for: Gfi1 and Gfi1b Repress Rag Transcription in Plasmacytoid Dendritic Cells In Vitro
Source: PLoS One. 2013 Sep 24;8(9):e75891. doi: 10.1371/journal.pone.0075891 (PMC3782466; doi:10.1371/journal.pone.0075891)

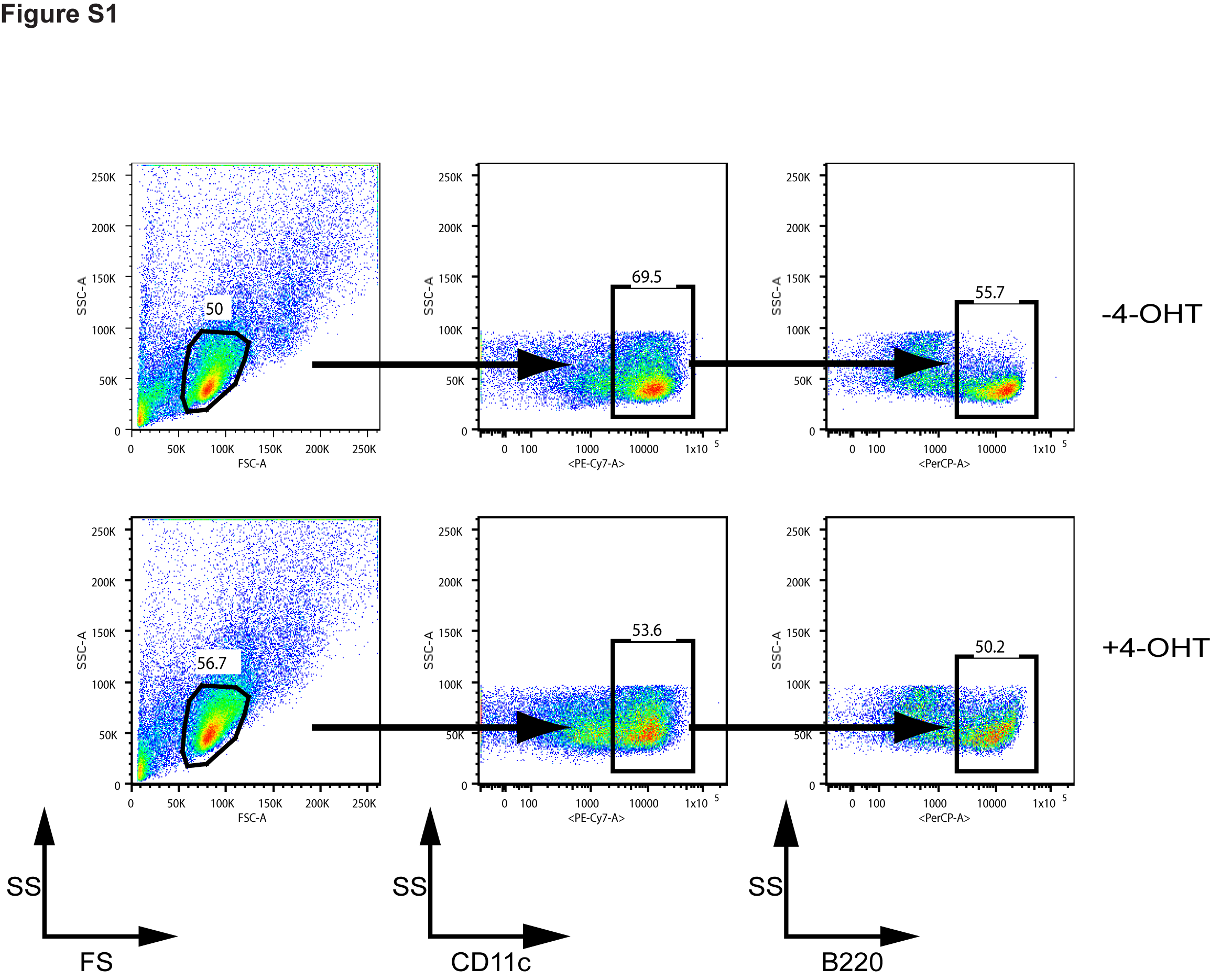

Supplement: Figure S1 — Gating strategy of pDC cultures. Representative gating strategy of flow cytometric analysis of pDCs derived from FLT-3L cultures. Cultures were untreated (top panel) and treated (bottom panel) with tamoxifen (4-OHT). Cells were gated on FS/SS, then CD11c+, then B220+. (TIF) [file pone.0075891.s001.tif]
