# Supplementary material for: Gfi1 and Gfi1b Repress Rag Transcription in Plasmacytoid Dendritic Cells In Vitro
Source: PLoS One. 2013 Sep 24;8(9):e75891. doi: 10.1371/journal.pone.0075891 (PMC3782466; doi:10.1371/journal.pone.0075891)
Supplement: Table S3 — Primer sequences used in this study. (DOCX) [file pone.0075891.s004.docx]

**Table S3. Primer sequences used in this study**

| **Assay** | **Primer name** | **Primer sequence** |
| --- | --- | --- |
| transcripts | Rag1 | CATTCTAGCACTCTGGCCGG |
| transcripts | Rag1 | TCATCGGGTGCAGAACTGAA |
| transcripts | E2A common | GGG GAA GCC ATC CTG AGG AGG |
| transcripts | E47 | CGG CGC TCC TTC TCC CGC TCC |
| transcripts | E12 | GGG ACA GCA CCT CAT CTG TAC TG |
| transcripts | IL7r | CGAGTGAAATGCCTAACTC |
| transcripts | IL7r | GCGTCCAGTTGCTTTCAC |
| transcripts | Pax5 | GTC CCA GCT TCC AGT CAC AG |
| transcripts | Pax5 | AAT AGG GTA GGA CTG TGG GCC |
| transcripts | SpiB | GAGGACTTCACCAGCCAGACC |
| transcripts | SpiB | GCGTAGGAGCAACCCCAGCAA |
| transcripts | Gata3 | TCGGCCATTCGTACATGGAA |
| transcripts | Gata3 | GAGAGCCGTGGTGGATGGAC |
| transcripts | Blnk | CAC CCC CCT GGA CAG CGA CAC ATC |
| transcripts | Blnk | CTG GGCTTACTGGGAAGTGTCTTGCTG |
| transcripts | Iga | TCA TAC GCC TGT TTG GGT CCC |
| transcripts | Iga | CCC TCA TAG AGA TTT TCA TCT TCA |
| transcripts | Notch1 | TGTTAATGAGTGCATCTCCAA |
| transcripts | Notch1 | CATTCGTAGCCATCAATCTTGTCC |
| transcripts | EBF | GCCTTCTAACCTGCGGAAATCCAA |
| transcripts | EBF | GGAGCTGGAGCCGGTAGTGGAT |
| transcripts | K05 | GCCCAAGCGCTTCCACGCATGCTTGGAG |
| transcripts | Ckappa | GTCCTGATCAGTCCAACTGTTCAG |
| transcripts | mu0 | GCCAAGGCTAGCCTGAAAGATTACC |
| transcripts | I-mu | TTCCAATACCCGAAGCATTTAC |
| transcripts | CH | ATGCAGATCTCTGTTTTTGCCTCC |
| transcripts | H-2 | CGATTACATCGCCCTGAACG |
| transcripts | H-2 | GCTCCAAGGACAACCAGAAC |
| transcripts | Irf8 | AGACGAGGTTACGCTGTGC |
| transcripts | Irf8 | TCGGGGACAATTCGGTAAACT |
| transcripts | Ccr9 | GTCTCAGTTCCCCTACAACTCC |
| transcripts | Ccr9 | CGGAATCTCTCGCCAACAAAA |
| transcripts | Dntt | GTGGCTTCCACGCCTTATGA |
| transcripts | Dntt | GGCTCTTCGAGTTGTTCCCAT |
| transcripts | Tcf4 | GCCTCTTCACAGTAGTGCCAT |
| transcripts | Tcf4 | TCCCTGTTGTAGTCGGCAGT |
| genotype | Gfi1 common | CAGTCCGTGACCCTCCAGCAT |
| genotype | Gfi1 WT/Floxed | CTGGGAGTGCACTGCCTTGTGTT |
| genotype | Gfi1 delete | CCATCTCTCCTTGTGCTTAAGAT |
| genotype | Gfi1b common | GGTTTCTACCAGTCTGGCCCTGAACTC |
| genotype | Gfi1b WT/Floxed | TACATTCATGCTTAGAAACTTGAGTC |
| genotype | Gfi1b delete | CTCACCTCTCTGTGGCAGTTTCCTATC |
